# Genetically determined intelligence and coronary artery disease risk

**DOI:** 10.1007/s00392-020-01721-x

**Published:** 2020-08-02

**Authors:** Ling Li, Shichao Pang, Lingyao Zeng, Ulrich Güldener, Heribert Schunkert

**Affiliations:** 1 Department of Cardiology, Deutsches Herzzentrum München, Technische Universität München, Lazarettstr. 36, 80636 Munich, Germany; 2 Deutsches Zentrum für Herz- und Kreislaufforschung (DZHK), Munich Heart Alliance, Munich, Germany

**Keywords:** Coronary artery disease, Intelligence, Educational attainment, Genetic association, Genetic risk score, Smoking, Obesity

## Abstract

**Background:**

Epidemiological studies have shown inverse association between intelligence and coronary artery disease (CAD) risk, but the underlying mechanisms remain unclear.

**Methods:**

Based on 242 SNPs independently associated with intelligence, we calculated the genetic intelligence score (gIQ) for participants from 10 CAD case–control studies (*n* = 34,083) and UK Biobank (*n* = 427,306). From UK Biobank, we extracted phenotypes including body mass index (BMI), type 2 diabetes (T2D), smoking, hypertension, HDL cholesterol, LDL cholesterol, measured intelligence score, and education attainment. To estimate the effects of gIQ on CAD and its related risk factors, regression analyses was applied. Next, we studied the mediatory roles of measured intelligence and educational attainment. Lastly, Mendelian randomization was performed to validate the findings.

**Results:**

In CAD case–control studies, one standard deviation (SD) increase of gIQ was related to a 5% decrease of CAD risk (odds ratio [OR] of 0.95; 95% confidence interval [CI] 0.93 to 0.98; *P* = 4.93e–5), which was validated in UK Biobank (OR = 0.97; 95% CI 0.96 to 0.99; *P* = 6.4e–4). In UK Biobank, we also found significant inverse correlations between gIQ and risk factors of CAD including smoking, BMI, T2D, hypertension, and a positive correlation with HDL cholesterol. The association signals between gIQ and CAD as well as its risk factors got largely attenuated after the adjustment of measured intelligence and educational attainment. The causal role of intelligence in mediating CAD risk was confirmed by Mendelian randomization analyses.

**Conclusion:**

Genetic components of intelligence affect measured intelligence and educational attainment, which subsequently affect the prevalence of CAD via a series of unfavorable risk factor profiles.

**Graphic abstract:**

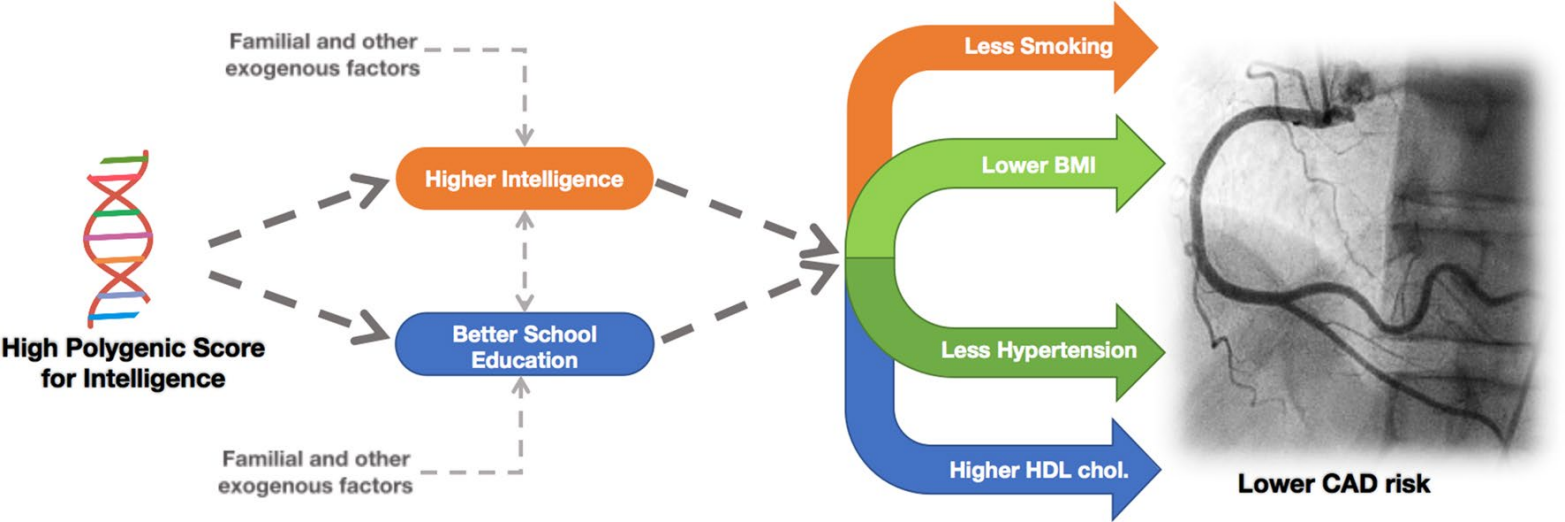

**Electronic supplementary material:**

The online version of this article (10.1007/s00392-020-01721-x) contains supplementary material, which is available to authorized users.

## Introduction

Epidemiological studies have shown an inverse association between intelligence score and risk of coronary artery disease (CAD) [1, 2]. Higher intelligence is also inversely associated with risk factors of CAD, like smoking and obesity [3–5]. Moreover, there is evidence for association between higher intelligence and longer educational attainment [6, 7] which may be an important mediator in reducing CAD risk [8]. However, the mechanisms linking higher intelligence with a decreased risk of CAD remain unclear.

Genome wide association studies (GWAS) have identified large numbers of genetic variants, typically single nucleotide polymorphisms (SNPs), associated with a wide range of complex traits providing opportunities of exploring the relationships between traits. Polygenic risk scores defined as sum of trait-associated SNPs weighted by effect size derived from large-scale GWAS measure the liability of individuals developing such traits [9, 10]. Thereby polygenic risk scores become an important genetic tool for studying association between traits [8, 11]. Two-sample Mendelian randomization (MR) is another genetic method of accessing causal relationships among traits which requires summary statistics of GWAS instead of full individual level genotype data and phenotypic measurements [12].

Savage et al. performed genome-wide association meta-analysis in 269,867 individuals and identified 242 SNPs independently associated with intelligence [13]. We used the statistics of these intelligence SNPs to perform both regression analysis of the individual-level polygenic score and two-sample MR analysis to study the association between intelligence and CAD risk, and to explore potential pathways from a higher genetic intelligence score to lower CAD risk.

## Methods

### Cohorts description of individual-level genotype data

Individual level genotype data were collected from ten case–control studies of CAD as discovery set [14–21]. All participants were of European descent, mostly from the Germany and UK. The replication set was from UK Biobank [22] which includes genotypes of 487,409 individuals derived from two different genotyping array platforms.

The data of UK Biobank were also applied to characterize interplay between intelligence and risk factors of CAD including body mass index (BMI), type 2 diabetes (T2D), HDL cholesterol, LDL cholesterol, hypertension, and smoking behavior. These traits were either self-reported or extracted from hospital episodes or death registries as reported by UK Biobank [22]. Intelligence scores were measured in UK Biobank through a 13-item verbal-numeric reasoning test designed to assess the ability of solving problems that require logic and reasoning ability, independent of acquired knowledge (field ID 20016). The total range of intelligence as measured by this score was from 0 to 13 arbitrary unit. Details of corresponding studies, data preprocessing and traits definition of data from UK Biobank are shown at Supplementary Notes and Table S1.

### Intelligence associated variants

Savage et al. performed GWAS meta-analysis of 14 independent epidemiological cohorts of European descent and reported 242 independent SNPs with genome-wide significant association (*P* < 5e–8) to intelligence scores [13]. We estimated effect size for each SNP from GWAS summary statistic table using method by Zhu et al. [23]. Details are shown at Supplementary Notes and Table S2.

### Statistics

The summary statistics of 242 independent SNPs of intelligence were applied to calculate the individual-level weighted genetic score of intelligence for each study. Firstly, each variant was given a value from 0 to 2 according to the presence of the intelligence allele in the imputed genotype data of each participant, which was then multiplied with the effect size of the variant on intelligence. For variants with missing genotypes in the imputed data, the reference allele frequency was applied. Then we summed these values of 242 variants for each participant as the polygenic score of intelligence, namely the genetic intelligence score (gIQ). Afterwards, the continuous gIQ was standardized into *z*-scores with mean of 0 and standard deviation (SD) of 1. By logistic regression analyses, we estimated effects of gIQ on CAD risk for each study separately. To control the bias due to population stratification or different genotyping platforms, the first two principle components for 10 CAD studies were added as adjustments of the regression model. In UK Biobank, because of more complex population structure, we employed top five principle components and array platforms for this data set. Lastly, the fixed-effect size meta-analysis was performed to estimate the combined effects across all CAD studies. Based on gIQ, all individuals were evenly separated into low, medium and high groups to study the distribution of cases and controls along with increasing gIQ.

Albeit the gIQ reflects intelligence at first place, the SNPs utilized in this score may be pleiotropic and thus affects other traits [24–26]. Seven of 242 intelligence SNPs were reported to be associated with educational attainment through a large scale GWAS cohort which detected 1271 education-associated SNPs [27]. We thus re-evaluated the association between gIQ and CAD risks after exclusion of 7 SNPs overlapping with educational attainment to estimate the direct effects of intelligence.

In UK Biobank, we estimated effects of gIQ on measured intelligence, educational attainment, risk factors of CAD including BMI, T2D, smoking, HDL cholesterol, LDL cholesterol and hypertension. Definitions of these traits are shown at Supplementary Notes and Table S3. Logistic regression was applied to binary traits like T2D, smoking, hypertension; and linear regression was for continuous traits like measured intelligence, educational attainment, BMI, HDL cholesterol, and LDL cholesterol. Top five principle components and array platforms were used as adjustment of regression models. We also studied phenotypical association of measured intelligence with educational attainment and CAD incidence in UK Biobank. Additionally, to avoid the genetic influence of education derived from genetic overlaps between intelligence and education, we re-estimated the effects of intelligence on CAD and its risk factors by eliminating seven overlapping SNPs [27].

### Two-sample Mendelian randomization analysis

Mendelian randomization (MR) is a method using genetic variants as instruments to study causal relationships between exposures and outcomes [28]. We introduced the multivariable two-sample MR analysis to investigate the direct casual effects of intelligence and educational attainment on CAD and its risk factors. This approach taking GWAS summary statistics as input measures effects of one standard deviation (SD) change in intelligence or educational attainment. As bias can be introduced in two-sample MR when using genetic consortia that have partially overlapping sets of participants, we selected consortia without overlaps. The GWAS summary statistics of CAD and its risk factors, educational attainment were acquired from CARDIoGRAMplusC4D (CAD) [17], GIANT (BMI) [29], TAG (smoking) [30], GLGC (HDL cholesterol, LDL cholesterol) [31], SSGAC (educational attainment) [27], and DIAGRAM (T2D) [32]. Elaborate description of these five studies can be found at Supplementary Notes and Table S4.

To address the influence of genetic overlaps between education and intelligence, we eliminated seven SNPs that are both associated with intelligence and educational attainment in MR analysis. Three MR methods including inverse-variance-weighted average (IVW) [33], MR-egger [34] and weighted median [35] were applied. Relationships significant (*P* < 0.05) in at least two of three methods were identified to be reliable and shown by IVW results. Lastly, sensitivity analysis of effects of intelligence and educational attainment on CAD were performed by excluding SNPs that were moderately associated with risk factors of CAD (*P* < 0.001) from intelligence SNPs and education SNPs respectively. Details are shown at Supplementary Notes.

## Results

### Effect of gIQ on the risk of CAD

Ten case–control studies of CAD with 16,144 CAD cases and 17,939 controls were included in this study. Majority of participants were from the Germany and UK. Individual-level genotype data and elaborate phenotype data from UK Biobank were used as validating set containing 20,310 CAD cases which were defined by either self-reported, or hospital episode and death registry data, and 406,996 controls. (Supplementary Notes and Table S1). For each cohort, we generated gIQ based on 242 SNPs reported to be genome-wide significantly associated with intelligence [13].

**Fig. 1:**
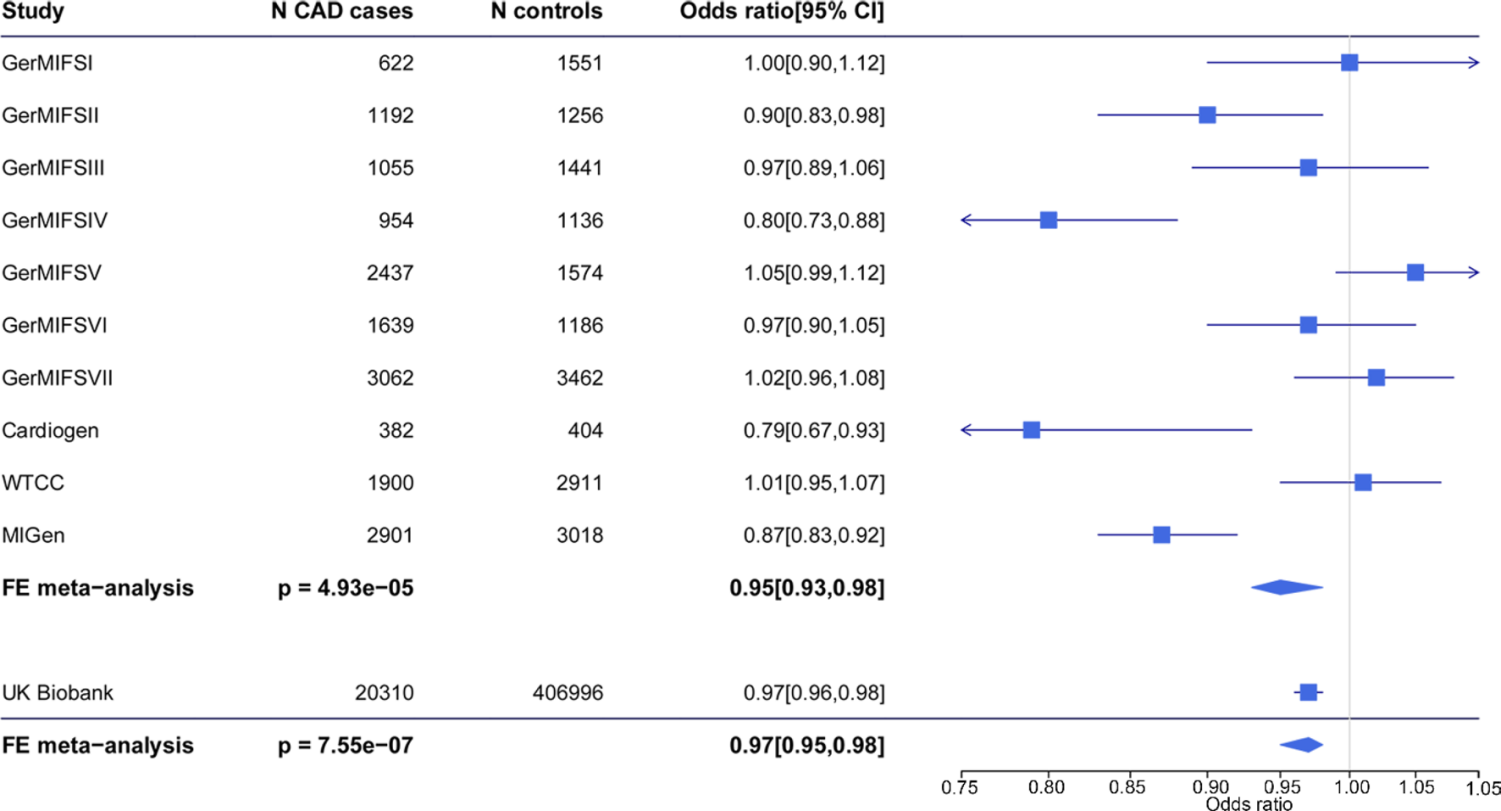
Association of gIQ and CAD risk. The genetic intelligence score was calculated in 10 case-controls studies of CAD and UK Biobank respectively. Logistic regression was performed to evaluate the association between gIQ and CAD risks in each study. Fixed-effect size meta-analysis was performed to combine all studies. Forest plot shows regression result in each study and the overall effect size. The gIQ was inversely associated with CAD risk

**Fig. 2:**
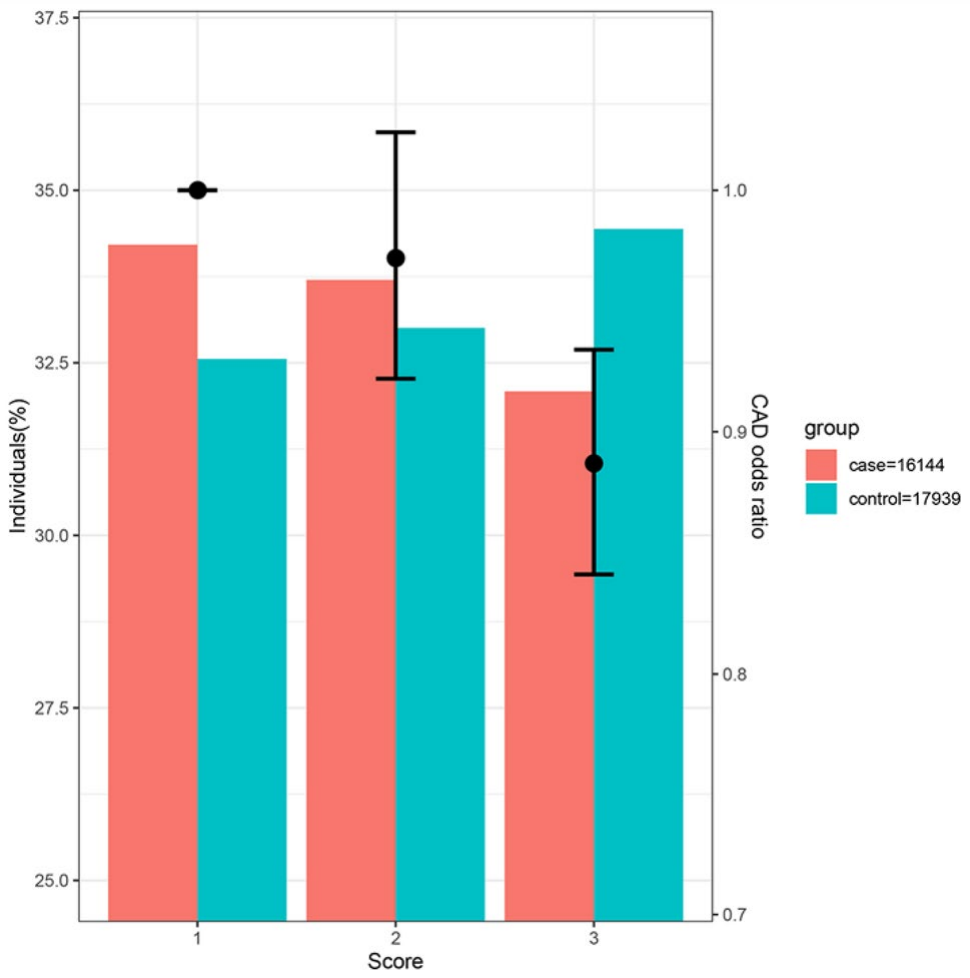
Distribution of cases and controls according to gIQ. Individuals in 10 CAD studies were evenly grouped into a low (score = 1), medium (score = 2) and high (score = 3) group according to their gIQ. The OR is incidence of CAD relative to low group. Risk of CAD decreases along the increases of gIQ

The score in participants of the 10 CAD studies was normally distributed (Fig. S1). Meta-analysis using fixed-effect size model indicated relative decrease of CAD risk by 5% (95% confidence interval [CI], 0.93 to 0.98; *P* = 4.93e–5) along with per 1-SD increase in gIQ (Fig. 1). When individuals were equally grouped into a low, medium and high group of gIQ, risk of CAD steadily decreased with an odds ratio (OR) of the high group vs low group being 0.89 (95% CI 0.84 to 0.93; *P* = 6.2e–6, Fig. 2).

**Fig. 3:**
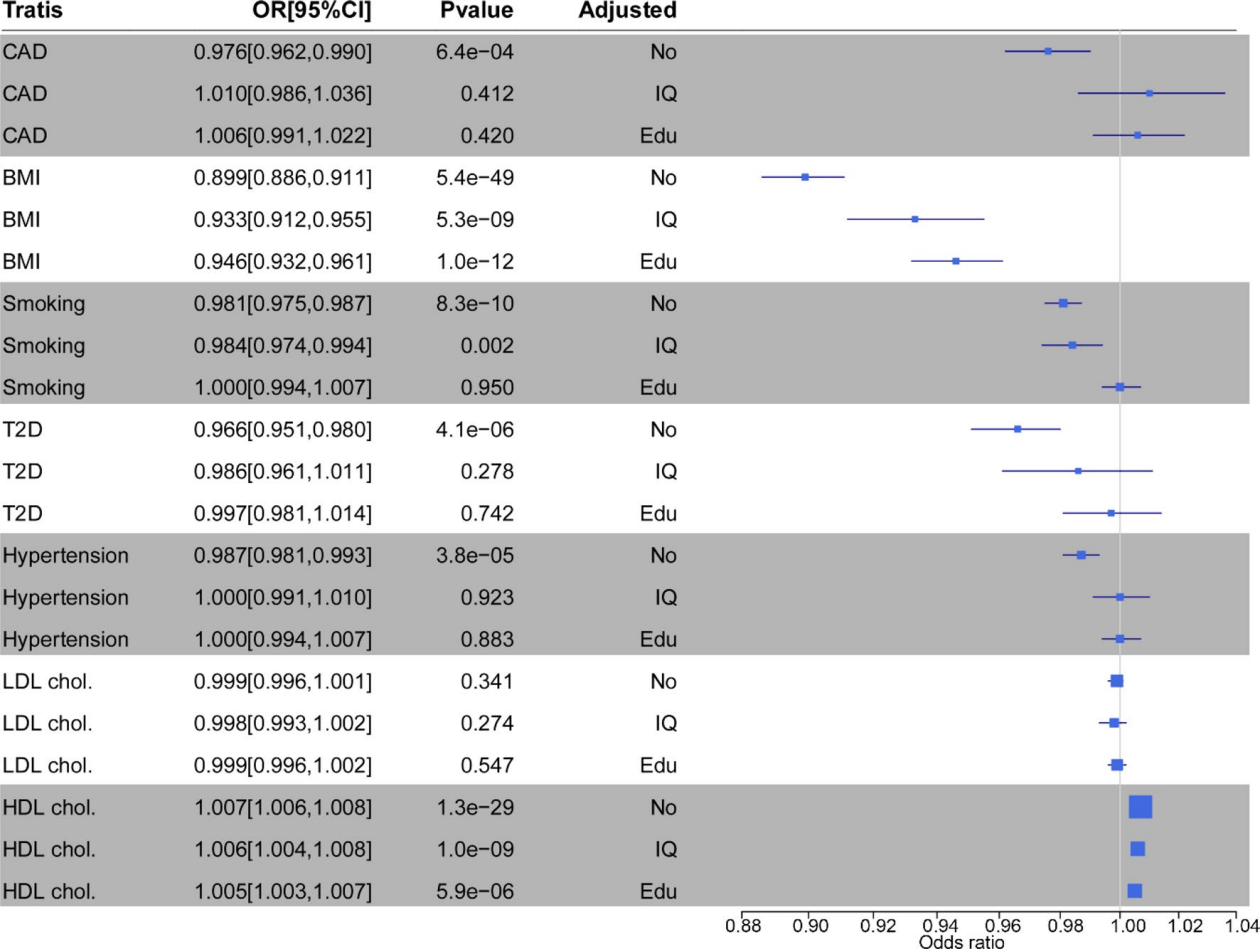
Associations of gIQ with CAD and it risk factors including BMI, smoking, T2D, HDL cholesterol, LDL cholesterol, and hypertension in UK Biobank. The OR for BMI is shown as logarithm of the linear regression coefficient. ‘Adjusted’ indicates the regression model between gIQ and trait after adjustment for measured intelligence (IQ), or length of school years completed (Edu), or neither of the two (No). The gIQ had inverse effects on BMI, T2D, smoking, and hypertension and a positive effect on HDL cholesterol. The association signals were largely attenuated by measured intelligence and educational attainment

Data from the UK Biobank confirmed the inverse association between gIQ and CAD risk with an OR = 0.97 (95% CI 0.96 to 0.99; *P* = 6.4e–4, Fig. 1). The risk of high gIQ group was 7% lower than the low gIQ group (*P* = 0.0005) in UK Biobank. As expected, the association between gIQ and CAD risk was abolished after adjustment for measured intelligence defining measured intelligence as an intermediary trait between gIQ and CAD risk (Fig. 3).

### Bidirectional association between intelligence and education

In UK Biobank, we found that 1-SD increase of gIQ increased measured intelligence score by 0.29 unit (*P* < 1e–10) and prolonged years spent in school by 0.45 year. In addition, one more year spent in school increased the measured intelligence score by 0.16 unit (*P* < 1e–10). Vice versa, one unit increase in measured intelligence prolonged years spent in school by 0.98 year (*P* < 1e–10). Both the measured intelligence and educational attainment had inverse effects on CAD risk. See results in Table S5.

### Effects of gIQ on risk factors of CAD

We next asked, in UK Biobank data, whether the association between gIQ and CAD risk was mediated by traditional risk factors of CAD, and whether such effects were dependent of measured intelligence and educational attainment. We found strong associations of gIQ with BMI, smoking, T2D, HDL cholesterol, and hypertension (Fig. 3). The effects of gIQ on CAD risk factors were largely attenuated after adjustment for measured intelligence or educational attainment (Fig. 3 and Table S6), suggesting that measured intelligence and educational attainment mainly mediated associations between gIQ and these risk factors. The analyses after removal of seven SNPs overlapping between intelligence and educational attainment obtained quantitatively and qualitatively similar effects of gIQ on CAD and its risk factors (Fig. S2).

We also studied the mediatory roles of these risk factors on the association between gIQ and CAD risk by applying them as adjustments to the regression model. Adjusting for individual risk factor or risk factors combined markedly attenuated association signal between gIQ and CAD risk (Fig. S3), indicating these risk factors were involved in mediating the association between gIQ and CAD risk.

### Mendelian randomization validation

**Fig. 4:**
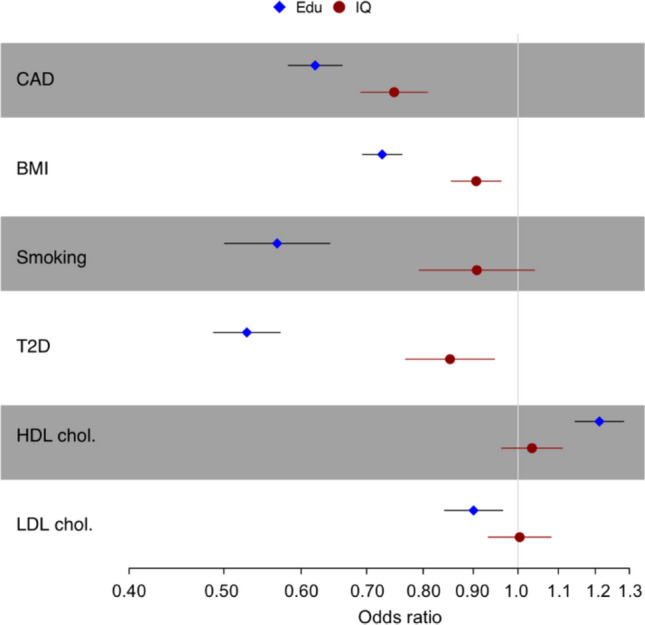
The result of MR analyses. Error bars indicate 95% confidence intervals around the estimated effects calculated using multivariable two-sample MR. The effects on outcomes for intelligence and educational attainment were generally in consistent directions. But the effects of educational attainment are quantitatively stronger than intelligence

To substantiate our observations, we performed multivariable two-sample MR analysis taking intelligence or educational attainment as exposures, CAD and its risk factors as outcomes. The estimates of the direct effects on outcomes for intelligence and education were generally in a consistent direction (Fig. 4). 1-SD increase of intelligence resulted in decrease of CAD risk by 25% (OR = 0.75; 95% CI 0.69 to 0.81; *P* < 1e–10), decrease of BMI by 0.1 kg/m^2^ (95% CI − 0.16 to − 0.14; *P* = 1.02e–3), decrease of T2D risk by 15% (OR = 85; 95% CI 0.77 to 0.95). A SD increase in the education years resulted in decrease of risk of CAD by 38% (OR = 0.62; 95% CI 0.58 to 0.66; *P* < 1e–10), decrease of BMI by 0.32 kg/m^2^ (95%CI − 0.37 to − 0.27; *P* < 1e–10), increase of HDL cholesterol by 0.19 mmol/L (95% CI 0.14 to 0.25; *P* < 1e−10), decrease of the risk of smoking by 43% (OR = 0.57; 95%CI 0.501 to 0.642; *P* < 1e–10), and decrease of T2D risk by 47% (OR = 0.53; 95%CI 0.49 to 0.57; *P* < 1e–10). The effects of educational attainment on CAD and its risk factors displayed the same direction as intelligence but were stronger in magnitude. See details at supplementary notes and Table S7.

Lastly, MR sensitivity analysis were performed for intelligence and educational attainment respectively. For intelligence, SNPs moderately associated (*P* < 0.001) with CAD (*n* = 5), BMI (*n* = 45), and HDL cholesterol (*n* = 5) were removed from intelligence SNPs. The sensitivity analysis showed 1-SD increase in intelligence to decrease the risk of CAD by 22% (OR for IVW method of 0.78; 95% CI 0.72 to 0.84; *P* = 5.6e–10). Same as intelligence, SNPs moderately associated (*P* < 0.001) with CAD (*n* = 13), BMI (*n* = 155), HDL cholesterol (*n* = 6), LDL cholesterol (*n* = 5), and smoking (*n* = 2) were removed from education SNPs. The sensitivity analysis showed 1-SD increase in education years to decrease the risk of CAD by 34% (OR for IVW method of 0.66; 95%CI 0.62 to 0.70; *P* < 1e–10). Results are shown at Table S8.

## Discussion

Epidemiological studies have revealed that increased intelligence correlates with reduced CAD risk [1, 2]. Consistently, our study shows that 1-SD increase of gIQ based on accumulated effects of genetic variants associated with intelligence, results in 5% decrease in the risk of CAD. The CAD risk in the high group of gIQ is relatively lower by 11% than in the low group. The observation was replicated in the UK Biobank. Interestingly, the inverse association got largely attenuated after adjustment for measured intelligence and educational attainment supporting the hypothesis that these traits play a role in modulating CAD risk.

Our study also shows the inverse effects of gIQ on health-related outcomes including BMI, smoking, T2D, hypertension, and a positive effect on HDL cholesterol, which are well-known for their influences on CAD risk [36–40]. Same as for CAD, these association signals appear to be largely mediated by measured intelligence and educational attainment. It can be concluded that these risk factors mediate the association between gIQ and CAD risk individually and collectively.

Our study confirms that intelligence and educational attainment are genetically and phenotypically associated with each other [6, 7]. Like in the present study, a recent study by our group states that educational attainment is inversely associated with CAD risk which appears to be mediated by risk factors such as BMI and smoking [8, 26]. Interestingly, our current study indicates that the effects of educational attainment on CAD and its risk factors are quantitatively stronger than respective effects of intelligence. All these findings indicate that improving educational attainment can have potential benefits in improving decision-making regarding health-relevant lifestyle factors and reducing risk of CAD and other health-related outcomes.

Polygenic risk score and two-sample MR are two genetic approaches of investigating association between traits. Compared with the traditional epidemiologic approach, the genetic approach is unlikely to be confounded by lifestyle or environmental factors as genotypes are stable over lifetime [11]. The utilization of genetic methods is limited, however, by false discovery because of horizontal pleiotropy, a phenomenon explained by the fact that variants may affect multiple traits through different pathways [9, 10]. The complex interplay of intelligence and educational attainment caused by their genetic roots limits a precise causal relationship between intelligence and CAD as well as its risk factors. In our study, we aimed to exclude genetic overlaps between intelligence and education to highlight putative causal effects of intelligence on CAD and its risk factors. Indeed, this notion was furtherly confirmed by MR analysis and the MR sensitivity analysis after excluding SNPs marginally associated with risk factors of CAD from intelligence (or education).

There are some limitations in our study. First, the intelligence SNPs utilized in this study were identified from a large GWAS meta-analysis based 14 independent epidemiological cohorts of European ancestry [13]. To avoid bias due to difference in population genetics, we restricted our analysis to cohorts from Germany, UK, and others of European ancestry. Second, there might be other health-related or socioeconomic factors that interplay with intelligence and CAD risk [26]. Specially, environmental exposures can be important confounders of association between intelligence and CAD risk. Third, the measured intelligence obtained in UK Biobank through a 13-item verbal-numeric reasoning test does not equal to real intelligence whose full scopes are unspecifiable. Moreover, educational attainment defined as years spent in schools in this study has a wide spectrum in various countries. Last, the two-sample MR analyses are likely to be biased if two studies contains overlapping participants or cohorts which are quite common in large-scale GWAS meta-analysis [41]. We tried best to choose studies that are of European ancestry and have minimal overlaps to avoid such bias in two-sample MR analysis.

**Fig. 5:**
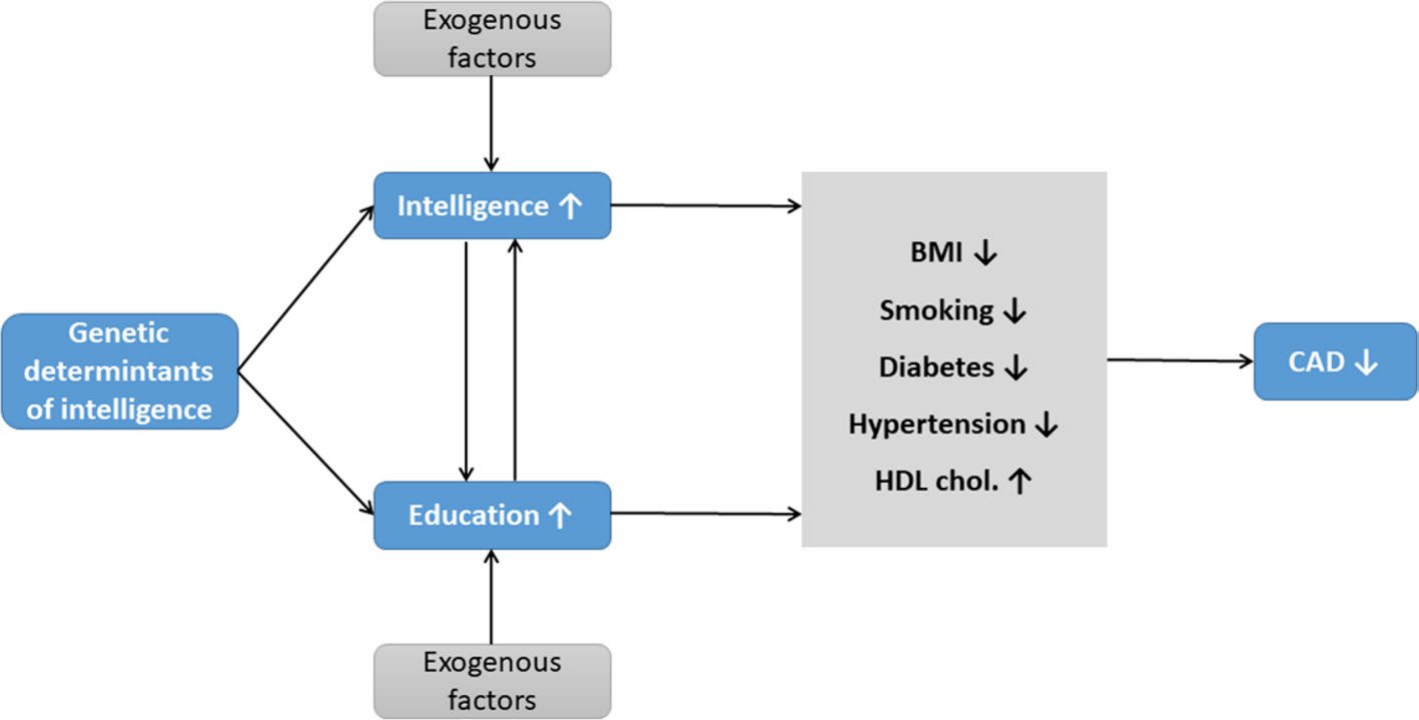
Pathway from higher gIQ to lower risk of CAD. Our study shows inverse effects of genetic determinants of intelligence on CAD and its risk factors including BMI, smoking, hypertension, and T2D and positive effects on HDL cholesterol. These association signals are mediated by measured intelligence and educational attainment, which two are bidirectionally associated with each other

In conclusion, using genetic approaches, we depicted a pathway from gIQ to CAD risk (Fig. 5). The higher gIQ is associated with the higher measured intelligence and longer educational attainment, both of which appear to reduce the prevalence of risk factors of CAD including BMI, smoking, T2D and hypertension, and increase HDL cholesterol, which in concert subsequently reduce the prevalence of CAD. Moreover, the effects of educational attainment on risk factors and CAD appear to be stronger than the effects of intelligence. Thus, repetitive campaigns throughout schooling may be worthwhile for preventive reasons as they may ameliorate the association between gIQ and unhealthy lifestyle.

## Electronic supplementary material

Below is the link to the electronic supplementary material.

Supplementary file1 (XLSX 54 kb)
